# Competition over guarding in the Arabian babbler (Turdoides squamiceps), a cooperative breeder

**DOI:** 10.12688/f1000research.6739.2

**Published:** 2016-02-04

**Authors:** Arnon Dattner, Amotz Zahavi, Avishag Zahavi

**Affiliations:** 1 Free environmental education in Nicaragua, SONATI, León, Nicaragua; 2 Department of Zoology, Tel-Aviv University, Tel-Aviv, Israel; 3 ARO The Vulcani Center, Bet-Dagan, Israel

**Keywords:** altruism, allofeeding, dominance, fitness, handicap, prestige, sentinel, signal

## Abstract

Observations on 12 groups comprised of two adult males and one adult female (some included one or two fledglings), tame, individually marked, Arabian babblers ( *Turdoides squamiceps)* in the rift valley in Israel revealed that the babblers compete to guard. The pattern of guarding and the way by which one sentinel replaces another reflect the dominance relationships within the group. The dominant (alpha) male guarded more than any other individual. It interfered with and replaced the guarding by the adult beta male more than it did with the yearlings. About one-third of the replacements occurred less than one minute after the sentinel had assumed guarding. Whereas the dominant often replaced its subordinates directly; subordinates hardly ever replaced their dominants directly. The alpha male often allofed the beta male during the replacement. Replacements and allofeeding of the beta males by the alpha males increased significantly during courtship, when competition over breeding was maximal, and dropped back to their previous level at the start of incubation, highlighting the competitive basis underlying the act of guarding. Competition over altruistic acts, as shown here for guarding, is not compatible with explanations based on the assumption that altruistic acts reduce the fitness (reproductive success) of the altruist. We suggest, in contrast, that by investing in guarding and by intervening in the guarding of its competitors, a babbler demonstrates and signals its quality and its control over its competitors, thereby increasing its prestige and consequently its direct fitness.

## Introduction

The issue of altruism is still a major question in evolution. Trivers (1985) defined altruism as an "act that confers a benefit on someone at a cost to the other *(the altruist).*" "…cost is measured by a decrease in reproductive success". Several theories have been posited to suggest that the altruist gains indirectly, among them are group-selection (including group augmentation), kin-selection and reciprocal-altruism. All these theories are based on the assumption that the altruistic act reduces the reproductive success of the altruist, and were developed in order to explain how altruism has persisted despite its supposed shortcomings. Zahavi (1977); Zahavi (1990); Zahavi (1995) and Zahavi & Zahavi (1997) contended that many altruistic acts can be explained as activities that signal the performer's claim to social prestige, and that increase rather than decrease the fitness of the altruist. Consequently, contestants would be expected to compete to perform the altruistic act. Such competition has already been shown in babblers for allofeeding and feeding at the nest by yearlings ( Carlisle & Zahavi, 1986); for mobbing of raptors ( Anava, 1992); in confrontations with neighbours ( Berger, 2002) and in allofeeding among adults ( Kalishov *et al.*, 2005).

Guarding, the activity of one or more individuals taking turns to watch over the group, is a very common phenomenon among group-living birds and animals (see Ridley *et al.*, 2013 for references). Many observations have confirmed that the presence of a sentinel allows the group to forage in comfort ( Hollén *et al.*, 2008; Ben-Mocha, 2013). However, that guarding helps the group does not explain why a particular individual may forego foraging or any other occupation, in order to guard.

In this paper we present data showing that the alpha and beta male babblers compete to perform the altruistic act of guarding. Other aspects of the phenomena of guarding are not discussed.

## Methods

Arabian babblers are thrush-size, group-living song birds ( Zahavi, 1989; Zahavi, 1990; Zahavi & Zahavi, 1997). The study took place at the Shezaf Nature Reserve, near Hazeva Field Study Center, in the Rift Valley, 30 km south of the Dead Sea, Israel (coordinates: 30_46¢N, 35_14¢E). The site is an extreme desert, summers are hot and dry. Mean winter rainfall amounts to 35 mm, but it may be as low as a few millimeters in dry years. This babbler population has been studied since 1971 ( Zahavi, 1989; Zahavi, 1990). Groups are composed of 2–20 individuals. The birds are tame, and observers can make close observations without disturbing them. The lineage and life history of most of the individuals is known from the time they were colour-banded as nestlings for individual recognition. The groups are resident and territorial. They maintain a strict age- and gender-dependent dominance hierarchy. Only the alpha male and female usually reproduce in the single nest, although sometimes more than one male may father the young and more than one female may lay in the common nest ( Lundy *et al.*, 1998).

All group members share in activities such as defending the territory against intruders, sentinel activities, mobbing, incubation, feeding and caring for the young. There is very little overt aggression, except among the very young. The birds spend most of the time in close proximity, and when not foraging often clump, play or allopreen.

### The population

Observations were carried out from August 2003 to April 2004, following four years of serious drought, in which reproduction was reduced from 180 nestlings and fledglings, ringed in 1997, to the low number of about 30 ringed in each of the years 2000, 2001 and 2002. As a result the population was old and very stable ( Zahavi, 1990), enabling us to select a large number of similar groups, each comprising two adult males (Alpha male, M _1_; beta male, M _2_) and one adult female (F _1_). Some of the groups included one or two independent youngsters fledged in 2003 ( Table 1). The males were not related to the females and thus competed for reproduction, except in one group (MTE), in which the beta male was the son of the breeding female. In another group (HNC), there was acute competition between father and son culminating in eviction of the father leaving a group of two. Observation of this group ceased following the eviction. Competition over breeding was also intense in the SAL group, in which M _2_ was observed to copulate with the female and on several occasions interrupted the copulation of the dominant pair by following them ( Perl, 1996).

**Table 1.** Group composition, ages and relatedness among the males.

Group	Age *, **					Relatedness
	M _1_	M _2_	F _1_	Y _1_	Y _2_	of M _1_ to M _2_
Main groups						
BOK	8.7	5.8	>3	0.8	0.4	uncle
HOR	8.8	4.6	>03	0.8		father
MTE	9.8	4.6	9.7	0.5	0.5	uncle
MZR	>8	>7	3.8	0.5		unknown
NAV	>9	5.8	>3			father
SAL	9.8	8.7	>3	0.9	0.9	brothers
SZF	>9	>1	9.7			not related
Auxiliary groups						
BOT1	9.5	6.8	6.8	0.8	0.6	not related
BOT2	6.8	0.8	6.8	0.6		not related
POL	7.6	1.6	2.5	0.5	0.5	uncle
TMR	8.8	6.8	3.5	0.4	0.4	brothers
HNC	>11	4.6	>03			father

*Age (years, on 1/1/2004). ** > before the age denotes years since the individual joined the study population as an adult.

All the birds in this study were colour-banded for individual recognition and tame to the extent that they readily accepted food from the hand of the observer. None of the groups inhabited a territory bordering directly on agriculture.

Data on group composition, their ages and relatedness among the males are presented in Table 1.

### Descriptions of guarding patterns

**Figure 1.**
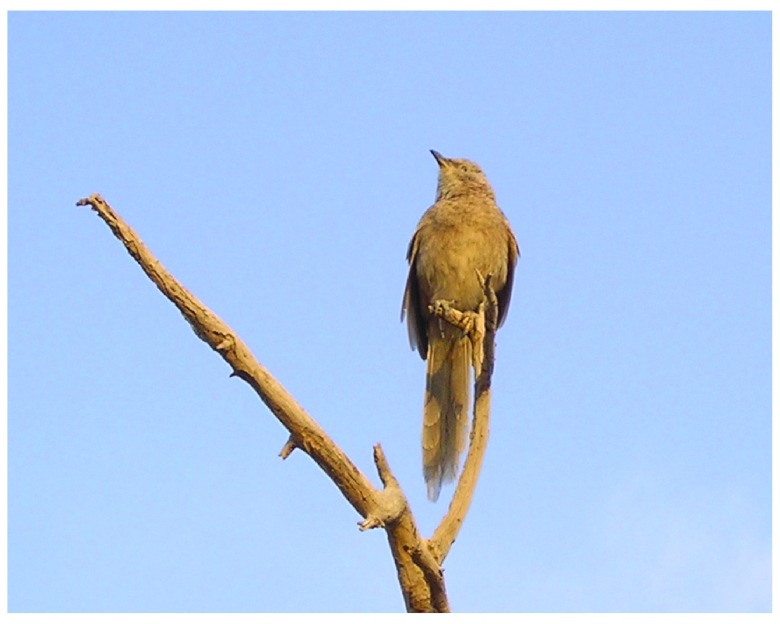
A dominant male Arabian babbler on guard on a high and exposed perch.

***a. Guarding.*** When the group is searching for food, or occupied in some other activity, one individual will stop feeding and climb onto a high place (e.g. a tree), inspecting the neighbourhood. It is usually easy to distinguish the sentinel from an individual that is in the tree for another reason, such as feeding, resting, auto-preening, etc. The height and extent of exposure of the sentinel depends on environmental conditions: in the darkness of dawn or during danger, it perches on the edge of the canopy. With increasing light it takes a more exposed perch, climbing to the top of the canopy, or often onto a dry branch devoid of leaves high above the canopy, from where it can see and be seen for a great distance. Many groups have a preferred guarding perch in their territory, highly exposed above the canopy ( Figure 1). Sometimes the sentinel flies directly to its guard post, but more often it climbs the tree, scanning around, then climbs to a higher and more exposed position and sometimes flies off to another tree. There is usually only a single sentinel, but when a predator or an alien babbler is sighted the whole group may perch for a while on the top of the tree. At the end of the guarding bout the sentinel either flies down directly or moves slowly into the canopy. A new individual starts its guarding bout at variable intervals after the previous sentinel had left its post. However, about 15% of the guarding bouts are started by the replacement of an active sentinel see below.

In contrast to many other species ( Bell *et al.*, 2010; Gaston, 1977), adult Arabian babbler sentinels do not emit regular calls, except for “alarm calls”, or shouts when interacting with distant babblers ( Sommer, 2011). Young sentinels, however, often produce soft sub-songs while they guard. These vocalizations attract attention to the youngster, advertising its activity to the group.

We do not know the proximate cause for a babbler to assume guarding and we do not discuss this issue here. However, we do know that the presence of foreign babblers in the territory increases guarding activity by the residents. The presence of an alien female in the territory greatly increases guarding by both the breeding female and the other females ( Ben-Mocha, 2013). Food availability too has a profound effect: on a rainy day little guarding was observed, but on the following day a maximum duration of almost 45 minutes/h was reached, probably due to the availability of a large number of insects that had drowned in the flooding ( Anava *et al.*, 2002).

***b. Replacements of sentinels.*** We observed several methods of sentinel replacement: it could be direct, with the replacer flying directly to the guarding bird, with or without some food item, and the previous sentinel then leaving its perch; or indirect, with the replacer taking up a guarding position on another perch. If the replacing bird was a subordinate, it often perched below the dominant or on another, usually lower, tree, and climbed to the final guarding position only after the dominant had vacated it. Eventually, mostly within one minute, one of the birds would abandon its position. In most cases, if the original sentinel did not leave its perch the second one retreated. The conflict between the two was often manifested by both of them nervously preening themselves.

***c. Allofeeding.*** Adult babblers sometimes allofeed ( Kalishov *et al.*, 2005). This often happens when the feeder seeks to replace the sentinel. We occasionally observed how a babbler that intended to replace another by allofeeding, first looked at the sentinel, and then started searching for a suitable food item. Upon finding one, it flew directly to the sentinel, fed it and usually replaced it. The sentinel either accepted the food (acceptance), sometimes crouching in a begging position (accepting like a fledgling), or rejected it. Finally it either did or did not leave its post. Often the sentinel, which may well be aware of the intention of the feeder - left its post before the feeder reached it. On rare occasions, when the sentinel did not accept the food item, the replacer (always a dominant) aggressively pushed the food into its beak. On several occasions a subordinate babbler that had just refused food offered by the dominant, immediately approached the observer to take a small crumb of bread, suggesting that despite being hungry it had refused to accept the "gift" from another babbler. Such interactions are not common but we have witnessed several over the years ( Zahavi & Zahavi, 1997).

***d. Social phases.*** We have defined three social phases: a. non-breeding; b. courtship, beginning when sticks are collected and lasting until the last egg has been laid; and c. incubation and feeding the young until independence. Breeding cycles were often aborted for various reasons.

### Observation and data collection

Every group was visited on average every 7–10 days. However, visits were not equally distributed – during mate guarding and egg laying a group could be visited daily until the first day of incubation, when frequency of visits usually dropped.

Observations started with first light, usually before the babblers had left their night-roost tree. For the first 2–3 hours they were followed without any interference by the observer. The data presented in this paper were collected during that period. Following these observations the babblers were offered some bread tidbits and water. This was done in order to induce allopreening, which was the main subject of the study ( Dattner, 2005). In one group (MTE) an entire day of observations without interference was conducted once a month. Of those days, only observations from the first three hours of the morning are included in the data presented here. We recorded the time of ascent and descent of the sentinel, its identity, the way by which it took up its perch, whether there was another sentinel at the time, whether it was replaced, the identities of the replacer and the replaced, type of replacement (direct or indirect or with allofeeding, acceptance or rejection of the food). The observations were written up on cards, noting the exact time and circumstances, and were later transferred to Excel spreadsheets. The social phase of the group in relation to the breeding cycle was also recorded. Table 2 presents the number of observation hours for each group, at the different social phases.

**Table 2.** Hours of observation * for the different groups: total and at the different social phases.

Group	Total	Social phase		
		Non- breeding	Courtship **	Incubation and dependent fledglings
Main groups				
BOK	107.3	36.9	37.8	32.6
HOR	46.5	23.3	14.7	8.5
MTE	64.9	45.5	11.9	7.5
MZR	64.3	30.2	21.2	12.9
NAV	56.3	24.8	27.3	4.3
SAL	128.2	24.5	85.7	18.1
SZF	65.8	14.1	42.1	9.67
Auxiliary groups				
BOT1	7.10	7.10		
BOT2	25.80		25.80	
POL	39.80		39.80	
TMR	14.58	6.33	8.25	
HNC	7.9		7.90	

*First three hours of the morning.

**Courtship phase: from collection of sticks to the start of incubation.

Weight was measured occasionally by other researchers who monitored the groups on alternate days, but not on our "observation days". The babblers were weighed in the morning as soon as they left the night roost. The birds were lured into mounting a scale (Moznei Shekel) by placing tiny tidbits of bread on it. The data presented in Table 3 are averages of several measurements taken over several months. A few individuals were not weighed because they either refused to mount the scale or were absent for some reason.

**Table 3.** Weight (g) * of M _1_, M _2_, and F _1_.

Group	M _1_	M _2_	F _1_
Main groups			
BOK	82.7	80.4	71.7
HOR	76.4		66.8
MTE	74.4	75.3	68.2
MZR			
NAV	72.0	77.0	
SAL	80.0	81.0	67.1
SZF	83.8	80.0	77.3
Auxiliary groups			
BOT1	85.0	80.1	81.8
BOT2	80.1		81.8
POL	76.4	79.1	71.0
TMR	72.2		70.0
HNC	74.8	75.1	73.0

*Averages of several dates. See text for details

The data presented in this paper summarize a total of 637 h of observations in the mornings without interference by the researchers: 212 h in the non-breeding phase, 322 h in the courtship phase (179 h during nest building and 143 h during mate-guarding), and 103 h while incubating and feeding the young. When not otherwise noted, numbers are averages per hour of observation.

Statistical analysis: Paired *t*-test was used to compare the behaviour of the alpha male, the beta male and the female in the group (when needed, Bonferroni-type adjustments for multiple comparisons were used). The *P* values are for a two-tailed alternative. Effect size calculations were also carried out using effect size calculators ( http://www.polyu.edu.hk/mm/effectsizefaqs/calculator/calculator.html).

### Results

Source data for the statistical analyses together with effect-size calculations Click here for additional data file. Copyright: © 2016 Dattner A et al. 2016 Data associated with the article are available under the terms of the Creative Commons Zero "No rights reserved" data waiver (CC0 1.0 Public domain dedication).

***a. Daily activity.*** Foraging for food was the main activity in the morning. Later in the day, when the birds were partly satiated, other activities dominated, such as allopreening ( Dattner, 2005), play ( Pozis-Francois *et al.*, 2004) and, mainly in summer, sleep. Activity in the afternoon was mixed. This pattern of activity is reflected in the number of guarding bouts during the day ( Supplementary material 1). In the first three hours the number of bouts was maximal, between 8–9 bouts per group per hour. Later, the number fell below 5, and averaged less than one bout per hour in the middle of the day (and down to zero in the hot hours of the summer). Sentinel activity was resumed together with the other activities in the afternoon, but did not reach the same level as in the mornings.

***b. Guarding.*** The mean (±SE) duration in which a sentinel was present in the morning was 22.75±1.3 ( *n* = 10 groups) minutes per hour of observation, meaning that even in the morning, at the time of maximal sentinel activity, a sentinel was present only for about one-third of the time. In every group there was great daily variability in the amount of sentinel activity, ranging from a minimum of 6.5 minutes to a maximum of 50.5 minutes per hour of observation ( Supplementary material 2).

**Figure 2.**
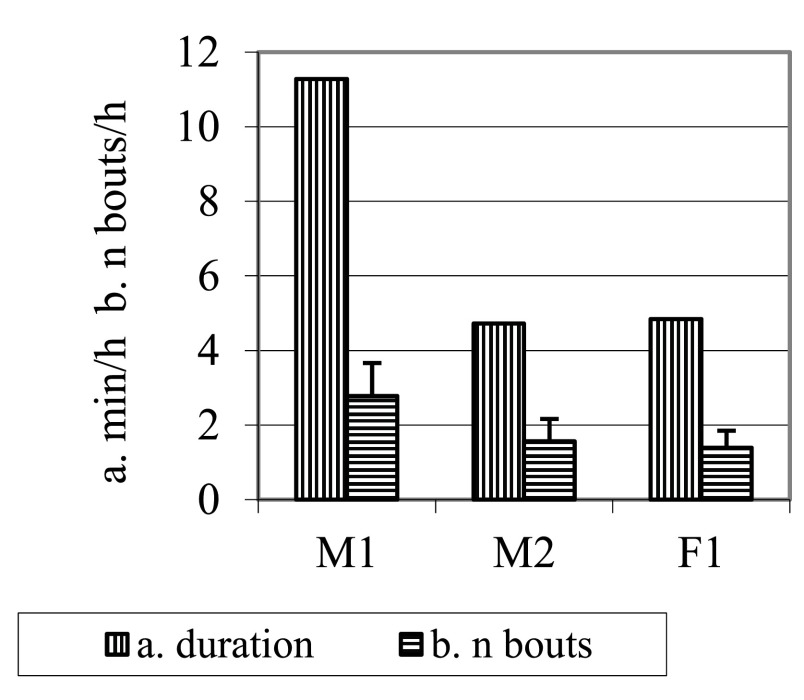
**a**. Duration of guarding (minutes/h) and **b**. number of guarding bouts/h by M _1_ by M _2_ and by F _1_.

The alpha male guarded for longer duration (a) and more frequently (b) than the other two adults ( Figure 2). (a). The mean duration of guarding was different between M _1_ (the alpha male) and M _2_ (the beta male) and between M _1_ and F _1_ (the female) ( *t*-test for paired observations, *t*_9_ = 5.157 *P* = 0.0012 and *t*_9_ = 5.642 *P* = 0.0006, respectively – *P* values are given after Bonferroni's correction). In both cases, the mean values for M _1_ were larger than for M _2_ and for F _1_. (b). The mean frequency of guard bouts was different between M _1_ and M _2_ and between M _1_ and F _1_ ( *t*-test for paired observations, *t*_9_ = 4.583 *P* = 0.0026 and *t*_9_ = 6.239 *P* = 0.0003, respectively – *P* values are given after Bonferroni's correction). In both cases, the mean values for M _1_ were larger than for M _2_ and for F _1_. Effect sizes (Es) for the differences in the duration of guarding and for the number of bouts between M _1_ and M _2_ were both large (see Dataset, Figure 2). In 80% of the 241 observation days the alpha male guarded more than any other individual in the group. Only on 20% of the days did the beta male or the female guard somewhat more than the alpha. This often happened when the latter was busy incubating ( Figure 11). There were no consistent differences in the number of bouts or in the duration of guarding between a beta male and a female.

**Figure 3.**
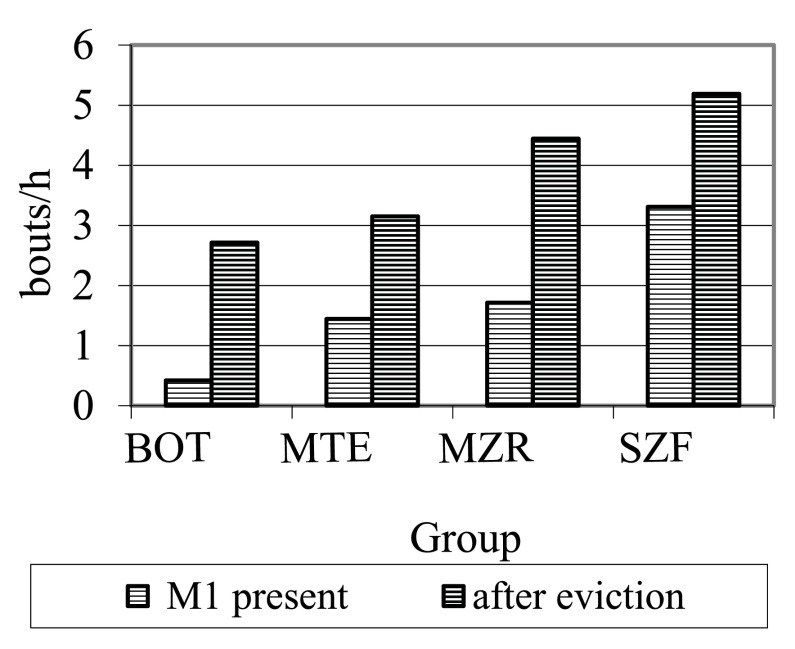
Guarding (bouts/h) by M _2_ before and after it evicted M _1_ in groups BOT1 MTE MZR SZF.

As soon as a subordinate male became dominant – following the eviction or disappearance of the alpha male – its guarding increased to the level of dominant ( Figure 3). (Before: 1.7211±1.1953 (mean±SD) After: 3.8738±1.1465. After minus Before: *t*-test for paired observations, *t*_9_ = 9.378 *P* = 0.0026). One could argue that the former M _2_ was now guarding against the former M _1_, which might still be present at the border of the territory. However, in group BOT1 the eviction of the M _1_ occurred early on in the observation period (with the group thus becoming BOT2), and the former M _2_ maintained a high rate of guarding until it was itself evicted. All other cases of eviction occurred towards the very end or after the end of the present study. 

**Figure 4.**
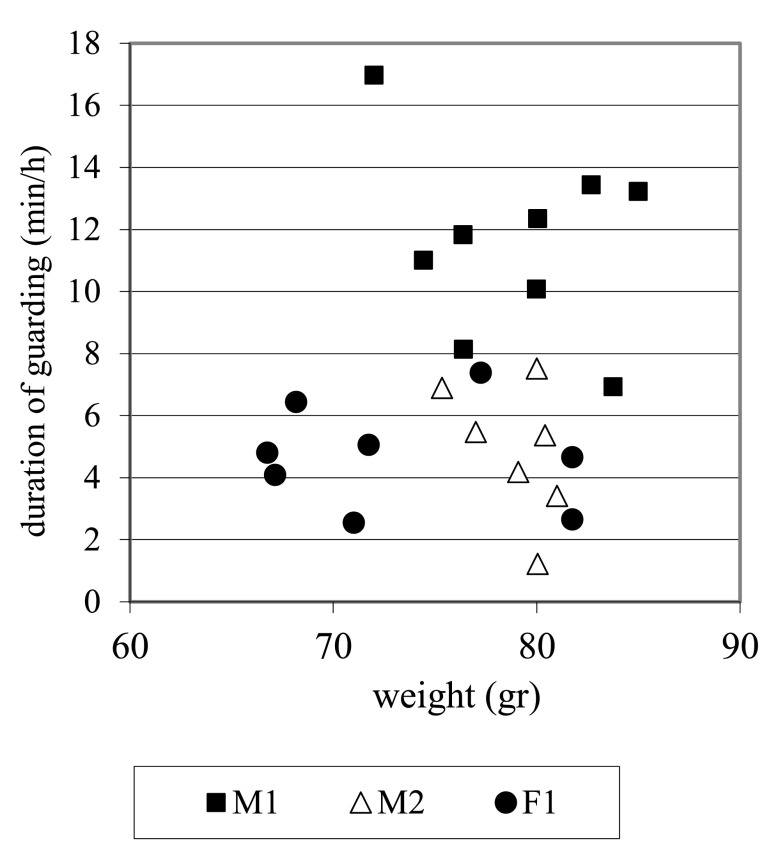
Relationship between a babbler's weight (g) and its duration of guarding (min/h).

***c. Relationship of guarding to body mass*** is presented in Figure 4. Within each category (M _1_, M _2_ and F _1_) there was no significant correlation between the babbler's weight and its guarding effort (Linear regressions: *R* = –0.310 for M _1_, *R* = 0.089 for M _2_, *R* = –0.116 for F _1_ – all are non-significant).

In five of the seven groups for which the weight of both males was known M _2_ was heavier than M _1_ ( Table 3) and in all of them M _1_ guarded much more than M _2_ (see Dataset, Figure 4). As soon as an M _2_ became dominant, its guarding increased to match that of the previous dominant ( Figure 3), without a corresponding change in its weight.

***d. Replacements of the sentinel.*** Replacements of a sentinel occurred on average about once per hour of observation. Thirty-percent of the replacements (32%±1.57, *n* = 14) occurred within 0–1 minutes after the original sentinel had started its guarding bout. When not interrupted, only about 10% (±1.24, *n* = 14) of the bouts lasted less than 1 minute. There was a significant difference between interrupted and uninterrupted bouts ( *P* < 0.01, *W* = 0, *n* = 14, Es: large.) ( Supplementary material 3).

**Figure 5.**
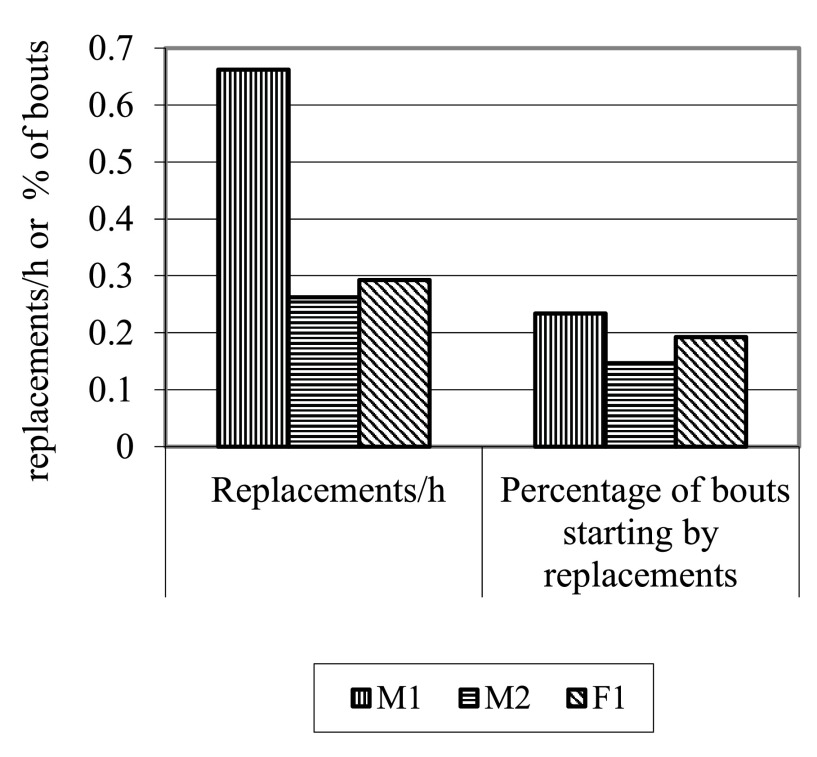
**a**. Number of replacements/h and **b**. percentage of guarding bouts that started by replacing an active sentinel.

The number of replacements/h as well as the percentage of guarding bouts starting with the replacement of an active sentinel is presented in Figure 5. Mean frequency of replacements by M _1_ is significantly larger than by M _2_ or by F _1_ ** ( *t*-test for paired observations with Bonferroni's correction, *t*_7_ = 4.829 *P* = 0.0038 and *t*_7_ = 7.657 *P* = 0.0002, respectively, Es: large), whereas mean frequency of replacements by M _2_ and by F _1_ are not significantly different.

In about 20% of its guarding bouts M _1_ started its session by replacing an active sentinel. M _2_ did so in 11% and an F _1_ in 18% of their guarding bouts respectively ( Figure 5b.) (Es: large).

**Figure 6.**
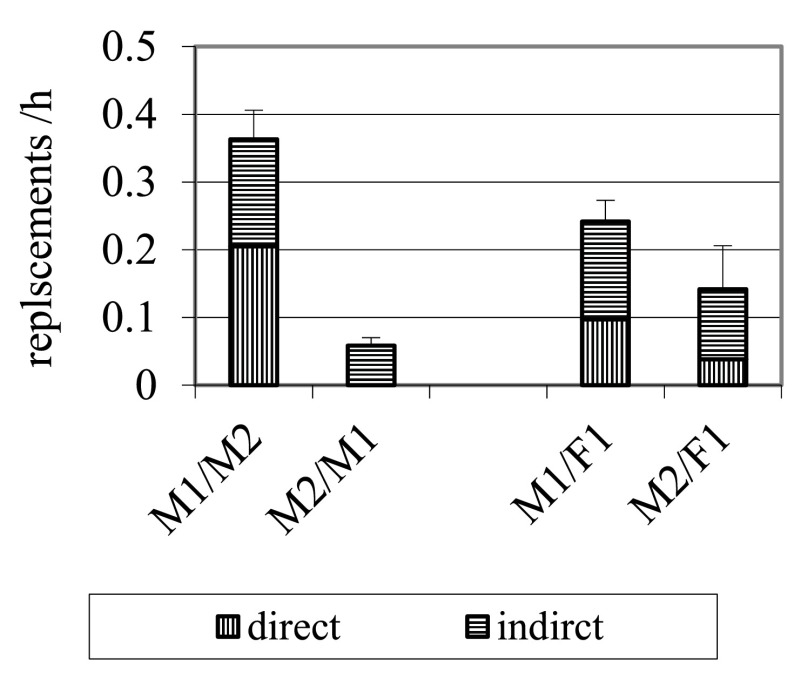
Direct and indirect replacements/h by M _1_ and by M _2_ (±SE for the total number of replacements).

**Figure 7.**
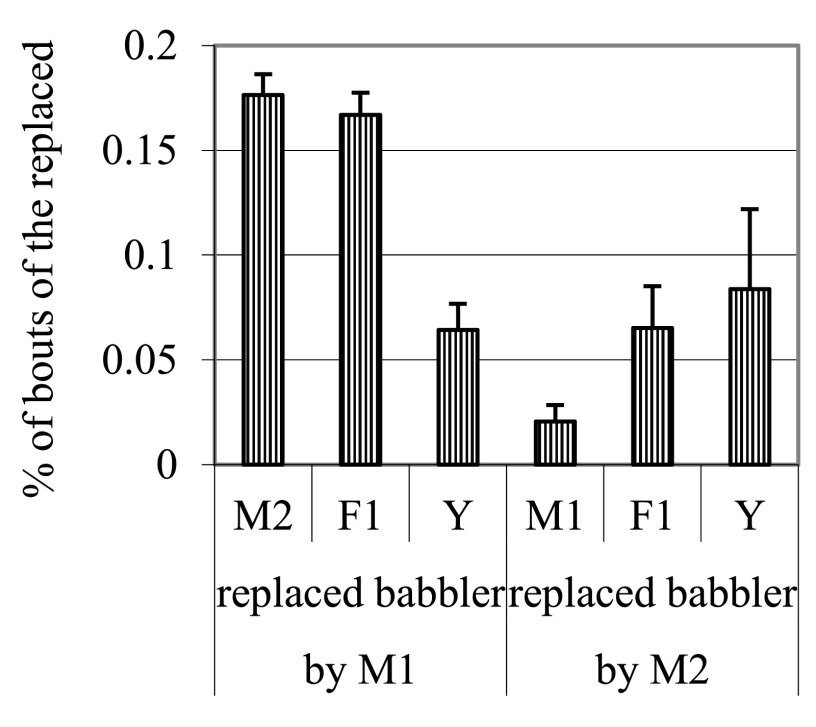
Replacements of adult or young sentinel by M _1_ or by M _2_, as percentage of the number of guarding bouts of the replaced babbler (groups with young only).

Figure 6 and Figure 7 show which individual replaced which other individual. Figure 6 presents the total number of replacements and number of direct and indirect replacements of M _2_ or F _1_ by M _1_ and of M _1_ or F _1_ by M _2_. In over 50% of encounters M _1_ replaced M _2_ directly. M _2_ never replaced M _1_ directly but they did replace the females directly (see Dataset, Figure 6). Total: M _1_/M _2_ more than M _2_/M _1_ ** ( *t*-test for paired observations, *t*_6_ = 7.994 *P* = 0.0002.). If we break the Total into its components (Direct and Indirect), we still have M _1_/M _2_ significantly more than M _2_/M _1_ for each component. (Direct: *t*_6_ = 5.943 *P* = 0.0010 Indirect: *t*_6_ = 8.357 *P* = 0.0002)

In Figure 7 replacements of the young are also shown. In this figure replacements are presented as a percentage of the guarding bouts of the babbler being replaced. This was done to compensate for the low number of bouts by the young. Only groups that included young birds are presented in this figure.

The figure clearly reveals that M _1_ replaced the sentinels much more often than M _2_. M _1_ also replaced its adult partners as sentinels much more often than it replaced the young inexperienced birds ( *p* < 0.05, *W* = 0, *n* = 5, Es: large). M _2_ rarely replaced M _1_ but it did replace females and young.

**Figure 8.**
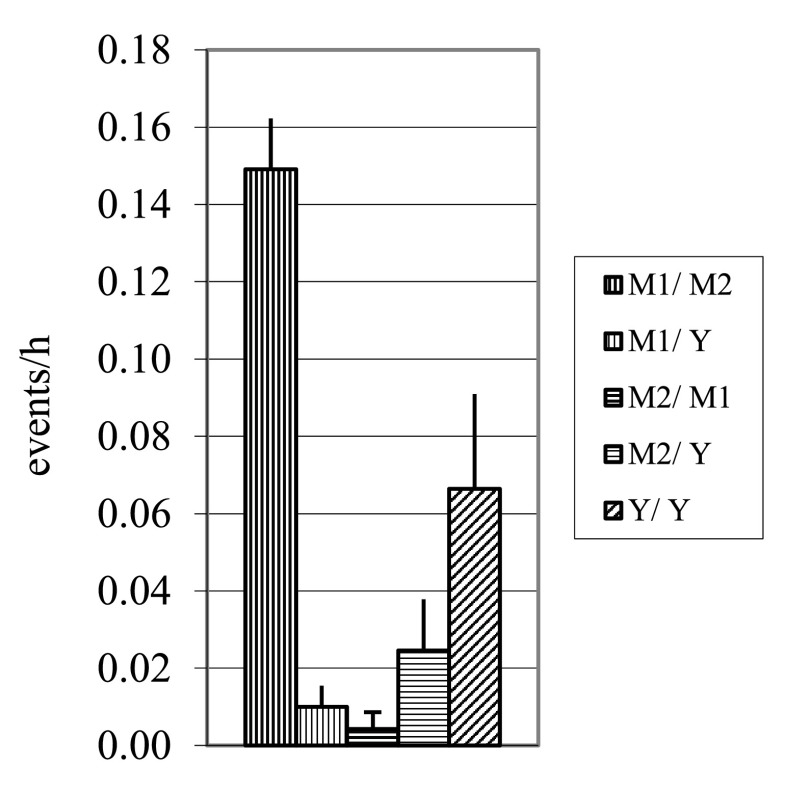
Replacing the sentinel by means of allofeeding (events/h±SE) among the males, among the males and the young, and among the young.

***e. Allofeeding.*** ( Figure 8, Table 4). On average, allofeedings during replacements were observed about once per observation session. Data in Figure 8 clearly show that M _1_ replaced M _2_ with allofeedings significantly more than it replaced and allofed the young ( *t*-test for paired observations, *t*_4_ = 9.418 *P* = 0.0007, Es: large), although the young were surely more in need of it. It is also apparent that the youngsters allofed each other more than they received from the adults. We never observed an M _2_ allofeeding an M _1_, although they do, on rare occasions, as was observed in other studies (unpublished report). M _2_ allofed both the females ( Table 4) and the young ( Figure 8). M _2_, and to a lesser extent the females, sometimes received the food in a crouching position, like fledglings. However, the food was also sometimes refused ( Table 4).

**Table 4.** Number of allofeedings by M _1_ or by M _2_ and type of acceptance. n/h= interactions per hour.; crouch= accepting as a fledgling (percentage of interactions); refusals (percentage of interactions).

Feeder	M _1_						M _2_			
Receiver	M _2_			F _1_			M _1_	F _1_		
group	n/h	Crouch	Refusal	n/h	Crouch	Refusal	n/h	no./h	Crouch	Refusal
BOK	0.17	53%	6%	0.13	14%	37%	0	0.05	15%	44%
HOR	0.19	23%	43%	0.25	36%	11%	0	0.09	16%	47%
MTE	0.17	15%	0	0.01	0	0	0	0		
MZR	0.17	0	13%	0.08	0	56%	0	0		
NAV	0.34	33%	0	0.13	0	0	0	0.16	22%	0
SAL	0.26	14%	21%	0.04	0	20%	0	0.10	24%	37%
SZF	0.60	44%	0	0.04	0	0	0	0.03	0	0

**Figure 9.**
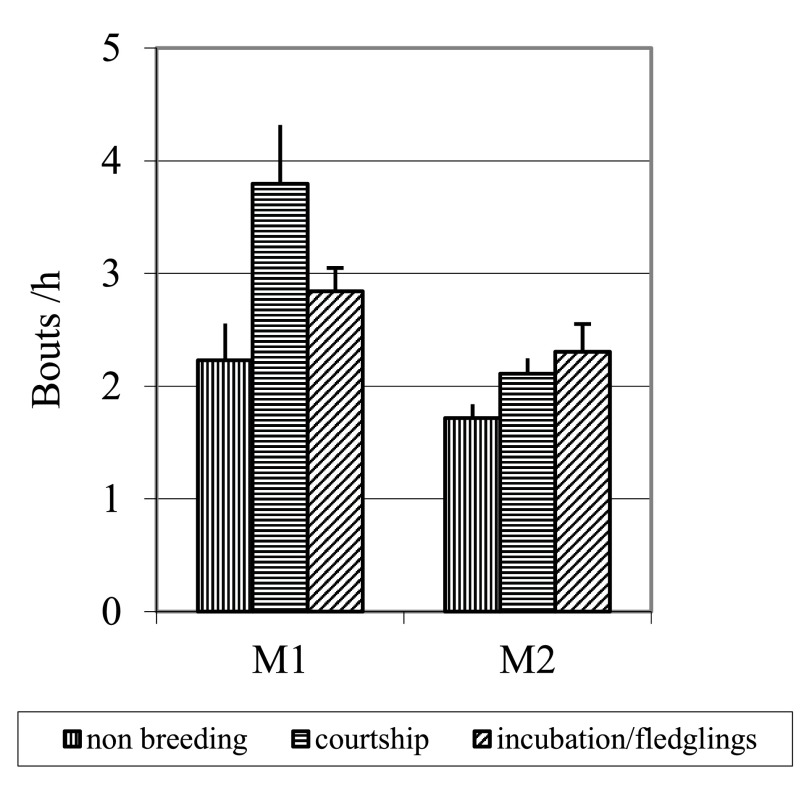
Effect of the social phase on the number of guarding bouts by M _1_ and by M _2_.

***f. Social phase.*** The breeding phase had a profound effect on all aspects of guarding, especially on that of the alpha male ( Figure 9– Figure 11). The number of guarding bouts by M _1_ increased significantly during the courtship phase but dropped back as soon as incubation started. Frequency of guarding bouts by M _1_ during Breeding is significantly larger than during Non-breeding: *t*_5_ = 4.413 *P* = 0.0139. Breeding vs. Incubation: *t*_5_ = 2.088 *P* = 0.1823, which is not significantly different (Bonferroni's correction has been applied). (Es: large). In some of the groups the M _2_ also slightly increased its guarding bouts ( Figure 9) in the courtship phase but it did not drop during incubation and feeding of the young.

**Figure 10.**
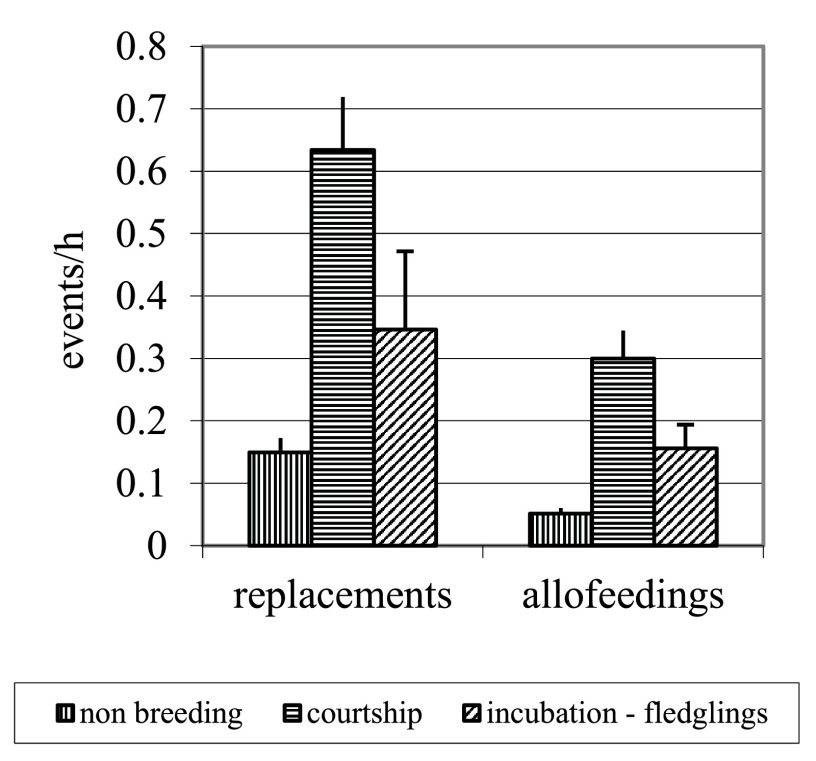
Effect of the social phase on replacements/h and allofeedings/h of M _2_ by M _1_ (+SE).

**Figure 11.**
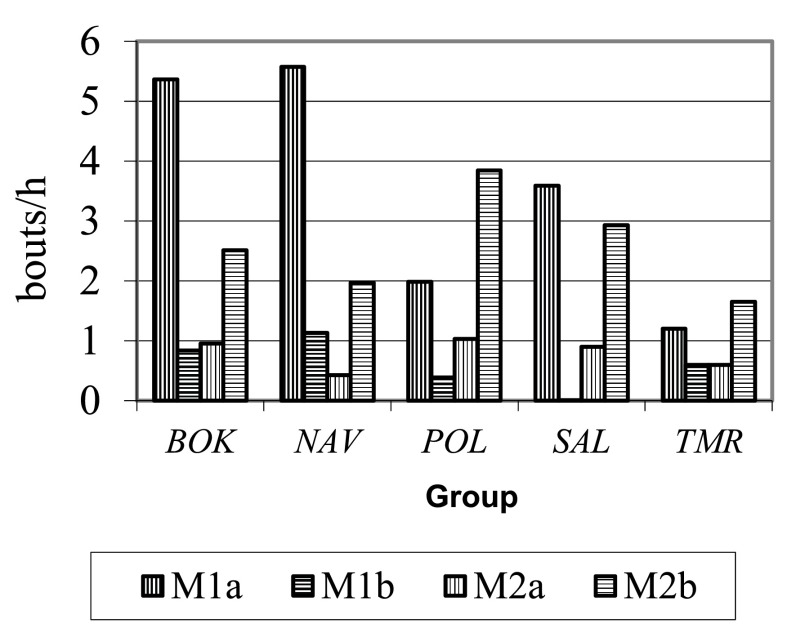
Guarding (bouts/h) by M _1_ and by M _2_ during a. mate guarding and b. the first day of incubation, in groups BOK, NAV, POL, SAL and TMR.

The number of replacements of M _2_ by M _1_ as well as the number of allofeedings of M _2_ by M _1_ also increased significantly during mate-guarding and then declined at the start of incubation: Replacements/h: Breeding vs. Non-breeding: *t*_5_ = 5.945 *P* = 0.0038, Breeding vs. Incubation *t*_5_ = 1.579 *P* = 0.3504.

Allofeedings/h: *t*_5_ = 5.458 *P* = 0.0056 Breeding vs. Non-breeding: Breeding vs. Incubation: *t*_5_ = 4.670 *P* = 0.0110.

During the first days of incubation the breeding-pair often monopolized incubation. On those days the guarding by M _1_ declined and those of M _2_ increased significantly in all groups ( *P* < 0.05 *W* = 0; *n* = 5 Figure 11).

M _1_ Mate Guarding vs. M _1_ Incubation: *t*_4_ = 3.740 *P* = 0.0403

M _2_ Mate Guarding vs. M _2_ Incubation: *t*_4_ = –6.043 *P* = 0.0076 (Please note a reversed direction).

M _1_ Mate Guarding vs. M _2_ Mate Guarding: *t*_4_ = 3.054 *P* = 0.0757

M _1_ Incubation vs. M _2_ Incubation: *t*_4_ = 3.848 *P* = 0.0367

(Bonferroni's correction has been applied). Note that the first, the second and the fourth comparisons (but not the third) are significant.

Different types of replacements: indirect, direct and with allofeedings can be visualized in this video clip: https://youtu.be/H_EdXAbRu2g

## Discussion

The data provided in this paper demonstrate that the alpha and beta males compete to guard. In addition to the alpha bird guarding more than any other individual, it also replaces and thus interrupts the guarding by other birds, especially that of the beta male, which competes with it over reproduction. The alpha male replaced the guarding beta male more than it replaced the young inexperienced birds ( Figure 7 and Figure 8). About one-third of guarding replacements took place less than one minute after a particular individual had mounted the guarding perch ( Supplementary material 3). This indicates that the sentinel that had just started its guarding bout was not yet hungry or tired, unlike what was suggested by Bell *et al.* (2010) for the pied babbler.

The dominant often replaced its subordinate directly ( Figure 6) by landing next to its perch, whereas the subordinate did not replace the alpha directly, but indirectly, by perching below it or taking a post on a lower tree, waiting for it to leave. The motivation of the beta male to replace the alpha was evident, for as soon as the latter had vacated its perch the beta male often climbed up to occupy the same perch. The alpha male also allofed the beta male during the replacement, sometimes forcing it to accept the gift, while the latter often reacted by fluttering its wings like a fledgling ( Figure 8, Table 4). The fact that all these behaviours increased significantly during courtship, when competition over breeding was maximal, and then declined at the start of incubation, after paternity had been decided and competition over immediate paternity was over ( Figure 9 and Figure 10), highlights the competitive basis underlying the act of guarding.

It should be stressed that most of the replacements were performed quite gently. The replacing bird often clumped with and sometimes allopreened the soon to be replaced sentinel for a few seconds or more before the latter left. However, when it refused to leave, it was sometimes pushed and even pecked at by the alpha male. As already noted, although overt aggression among adult males within the group is rare, aggressive replacements do occasionally occur.

Wright *et al.* (2001a ,b ,c) contended that a direct relation exists between a babbler‘s mass (weight) and the extent of its guarding: “Overall, body mass explained much of the variation in individual sentinel effort both within and between birds”, and “…found relatively little evidence that individuals compete for the chance to act as sentinels”.

Our data not only clearly contrast these statements, but some of the data presented in the figures of Wright *et al.*, contradict their own statements. They obtained a positive regression of mass vs. guarding effort ( Figure 4 of Wright *et al.*, 2001b) due to the inclusion of guarding by young immature group members, which were both lighter in weight and guarded much less than the adults. Indeed, the upper part of their figure differs little from our Figure 4, and clearly shows that within each of the upper three social categories (M _1_, M _2_, and F _1_) there is no correlation between body mass and guarding. In another study ( Wright *et al.*, 2001c), the average body mass of the beta males was given as greater than that of the alpha males ( Figure 3 of that study); but in eight out of the ten control days the sentinel effort by the alpha male was higher than that of the beta (their Figure 2, see also Figure 1 of Wright *et al.*, 2001b). Five of our beta males were heavier than the alpha males in their groups ( Table 3), but guarded much less than the alpha. In yet another paper, Wright *et al.* (2001a) state that "Change-overs between sentinels rarely revealed any social context"; but Figure 3 of the same study reveals large differences in change-overs between the alpha and beta males. Moreover, it has frequently been shown that supplementing the babblers with food greatly increased sentinel activity ( Bell *et al.*, 2010; Cordovi, 1988, unpublished report; Wright *et al.*, 2001b), suggesting that food availability constitutes a constraint on guarding, especially in the desert. However, except for the case of artificial differential feeding of one particular individual for single days ( Wright *et al.*, 2001b), food availability for the group did not alter the relative extent of guarding by the different hierarchical categories.

Wright *et al.* (2001a) also suggested that in large groups the load of guarding per bird was reduced. This argument was based on the division of the total time when a sentinel was present, by the number of birds in the group. However, our data show that the time that a babbler spends guarding depends mainly on its place in the hierarchy and other social and ecological aspects (including food availability), rather than on the mean number of birds in the group. Guarding by the maturing young does extend the overall period in which a sentinel is present ( Supplementary material 2), but it does not reduce the “load” of any particular individual.

Wright *et al.* (2001b) concluded that “...sentinel behaviour in Arabian babblers appears entirely consistent with recent (Bednekoff' *s*) models of selfish state-dependent sentinel behaviour”. Bednekoff (1997); Bednekoff (2001) proposed a model suggesting that "the sentinel position is the safest place to be… when no other group member is on guard" If that were indeed the case, why replace that sentinel? In contrast, in a recent paper, Ridley *et al.* (2013) found that in the pied babbler the sentinel is at a greater risk of predation and further from cover than the foragers. Our observations (unpublished report) suggest that this is the case also for the Arabian babbler.

Why did the beta males guard much less than the alpha males? In our groups the beta males were all mature and not much younger than the alphas in their group ( Table 1). Whenever they had the opportunity they guarded as much as the alphas. During the first days of incubation the breeding pair monopolize the incubation. Later on the beta male as well as the young may share in the incubation. In the few groups that were observed on the first day of incubation, the alpha male spent most of its time incubating rather than replacing the beta male. Consequently, the beta male increased its guarding almost to the average level of the alpha on other days ( Figure 11). One could argue that M _2_ was compensating for the absence of M _1_ – however, the large day-to-day fluctuations in the total amount of guarding ( Supplementary material 2) are not compatible with the notion that there is a certain daily "quota" of guarding. Indeed, in most cases, on days in which the alpha bird reduced its guarding - it still guarded more than any other individual in the group. The large increase in guarding by the beta male following its eviction of the alpha ( Figure 3) also indicates that the beta male's previous lower extent of guarding was not due to incompetence, or laziness or lack of desire to guard. We suggest rather, that guarding by the beta male was restricted by the alpha male.

Many observations have confirmed the fact that the presence of a sentinel allows the group to forage in comfort ( Hollén *et al.*, 2008 and references cited therein; Ben-Mocha, 2013). However, this does not explain why a particular individual will feel obliged to stop feeding, or any other activity, and stand guard over the group. Our calculations show that even in the morning, at the time of maximal guarding activity, a sentinel was present only for about one-third of the time. Why should a babbler choose frequently to replace an active sentinel rather than to start guarding when no other individual is guarding, and thus extend the overall time during which a sentinel is present? That the alpha male guards more than any other member of the group, and often replaces a subordinate as soon as the latter seeks to begin guarding, suggests that the primary concern of the alpha male is to demonstrate that it is the one that is performing the altruistic act, and that it can control the beta male and replace it whenever it wishes.

Anava (1992) studying mobbing of raptors in Arabian babblers, observed a similar competition among the males as well as among the females. In most groups the alpha male participated more than any other individual in all aspects of mobbing, and interfered with the mobbing of other group members, mainly that of the beta male; except in one group in which there was a single male and three adult females that competed over breeding. In that group the alpha female participated in the mobbing and guarding more than the male and interfered in the mobbing by the other females.

Competition over altruistic acts was also described in babblers with regards to: allofeeding and feeding at the nest by yearlings ( Carlisle & Zahavi, 1986); confrontations with neighbours ( Berger, 2002); and allofeeding among adults ( Kalishov *et al.*, 2005). All these altruistic activities confer benefit on the group and require investment by their performers. 

Competition makes sense if the contestants gain from winning. Any sentinel, not necessarily the alpha male, will satisfy the need of the group for a guard. If the reason for guarding is solely for the benefit to the group, why should a dominant not let a willing subordinate individual guard? We suggest ( Zahavi, 1990; Zahavi & Zahavi, 1997) that by investing effort in guarding and by intervening in the guarding of its competitors, a babbler demonstrates and signals its general ability and its control over its competitors.

Group members are aware of the presence of the sentinel, because in its presence they can feed and move about with greater ease; they know who is guarding ( Bell *et al.*, 2010) and are also attentive to the replacements and how these replacements are carried out. By guarding and by replacing a competitor, a babbler thus retains or increases its own prestige and reduces that of the competitor. We have suggested previously ( Zahavi, 1995; Zahavi, 2008) that this investment constitutes the handicap that proves the honesty of the claim to prestige.

Why should a babbler care so much about its prestige? High prestige provides the individual with a greater share in the group's resources. Prestige functions like an invisible "peacock's tail": it attracts collaborators and deters rivals. Zahavi & Zahavi (1997) suggested that whereas rank is stable, the prestige of an individual changes constantly. Every individual in the group, including the females and the very young, has and cares about its own prestige, especially in relation to those closest to it in the hierarchy. 

In more general terms, because acting altruistically may confer prestige on the performing individual, altruism can substitute for other means of obtaining prestige, such as overt aggression or wasteful display. This is especially important in such closely-knit social groups as the Arabian babblers. Threats and aggression can easily turn into fights that may result in the killing or eviction of the loser. The loser, if it survives the fight, becomes a refugee, while the winner loses a partner in the defense against its neighbours.

An altruist gains directly from the investment of performing altruistic acts, and the competition increases the likelihood that there will usually be a willing candidate to take upon itself the duty of the sentinel. Members of a social group are attentive to such acts because they both benefit from them and gain information regarding the social relationships among other members of the group. This may explain why altruistic acts are so common among animals living in cooperative groups.

## Ethics

The birds were banded and tamed under a permit from the Israel Nature and National Parks Protection Authority. No other permits were required.

## Data availability

The data referenced by this article are under copyright with the following copyright statement: Copyright: © 2016 Dattner A et al.

Data associated with the article are available under the terms of the Creative Commons Zero "No rights reserved" data waiver (CC0 1.0 Public domain dedication). 

*F1000Research*: Dataset 1. Source data for the statistical analyses together with effect-size calculations., 10.5256/f1000research.6739.d97780 ( Dattner *et al.*, 2015).

## Supplementary materials

Supplementary material 1. **Guarding (bouts/h, per group) throughout the day**. Figure and source data for MTE group.

.

Supplementary material 2. **Duration of guarding (min/h, in the first three hours of the morning) by different members of SAL group on observation days in February 2004 (breeding phase)**. Figure and source data for SAL group.

.

Supplementary material 3. **Brief bouts (0–1min).** A. As a percentage of uninterrupted guarding bouts or B. Bouts replaced 0–1 minutes after the start of the bout, as a percentage of total replacements of the replaced babbler.
