# Supplementary material for: Competition over guarding in the Arabian babbler (Turdoides squamiceps), a cooperative breeder
Source: F1000Res. 2016 Feb 4;4:618. Originally published 2015 Aug 24. [Version 2] doi: 10.12688/f1000research.6739.2 (PMC4706064; doi:10.12688/f1000research.6739.2)
Supplement: Peer review history

# Peer review: Referee response for version 2

**DOI:** 10.5256/f1000research.8394.r10064

**Published:** 2016-02-05

**Authors:** Uzi Motro

**Affiliations:** 1 Department of Ecology, Evolution and Behavior, Hebrew University of Jerusalem, Jerusalem, Israel

I approve the revised version, without reservations.

I have read this submission. I believe that I have an appropriate level of expertise to confirm that it is of an acceptable scientific standard.

# Peer review: Referee response for version 1

**DOI:** 10.5256/f1000research.7238.r11476

**Published:** 2015-12-09

**Authors:** Raghavendra Gadagkar

**Affiliations:** 1 Centre for Ecological Sciences, Indian Institute of Science, Bangalore, India

The evolutionary origins and maintenance of altruism are indeed major unsolved problems in the study of animals and humans. Most people attempt to find ways in which altruistic behaviour, which is believed to be detrimental to the fitness of the actors, is nevertheless favoured by natural selection. Zahavi and his co-workers on the other hand, have consistently pursued the idea that so-called altruistic behaviour is actually beneficial to the actor. This is because the very fact that the altruist is willing (and capable) to undertake costly altruistic behaviour increases his prestige and therefore his access to mates.

In this paper the authors attempt to provide support for their idea (often refereed to as the Handicap Principle), by showing that males of the Arabian babbler compete with each other to have the opportunity to undertake presumably costly, altruistic sentinel or guarding behaviour. Such a claim has been made by this group before but has been criticised by others who were either unable to find such pattern in sentinel behaviour of this species or argued that the observed pattern can be explained by other means. It may be very hard to conclusively demonstrate that males do or do not compete to perform sentinel behaviour or that all other explanations have been ruled out. The best way therefore to test the Handicap Principle in this case is to measure the actual costs and benefits of sentinel behaviour. This has not yet been done and is perhaps hard to do. Thus until such costs and benefits can be measured, we continue to rely on understanding and explaining the patterns of sentinel behaviour. In such a situation, wide acceptance of the Handicap Principle will necessarily depend on other researchers finding evidence in support of it. More research, more data and reiteration by the same authors and their associates are unlikely to be sufficient.

I would therefore argue that this is treated as an open question and researchers finding evidence for and against the Handicap Principle should have the opportunity to publish their findings and their interpretations, as long as they are scientifically rigorous. In this spirit I support the dissemination of this paper but to make it scientifically rigorous I recommend that the authors revise their manuscript in response to my comments below.

 

1. Introduction, last paragraph: If the authors wish to argue that altruistic acts are not actually altruistic, they should not continue to say “altruistic act of guarding”. Perhaps they should something like “supposedly altruistic act of guarding”.
2. Under the heading “Descriptions of guarding patterns a. Guarding”, the authors state that: “It is usually easy to distinguish the sentinel from an individual that is in the tree for another reason, such as feeding, resting, auto-preening, etc.” The authors should say clearly how to make such a distinction between a sentinel and a bird that is in the tree for another reason, so that other researchers can try to make the same distinction and it does not remain only a tacit knowledge of the present authors. I first assumed that the few sentences that follow this describe how to make the distinction but I am not sure of that. If that is indeed so, the authors should be explicit about it. More generally speaking, many things that Zahavi and his co-workers ‘know’ is a kind of tacit knowledge to which outsiders have no access. For Zahavi’s ideas to become more widely acceptable, they will have to make the effort to describe the sources of their knowledge and the reason for their convictions more transparent and thus allow others to enter into the same knowledge space.
3. In the same section at the end the authors mention that “but on the following day a maximum duration of almost 45 minutes/h was reached, probably due to the availability of a large number of insects that had drowned in the flooding (Anava *et al,* 2002)”. It is not clear why “high availability of insects” should increase guarding. Please explain.
4. Under the heading “Descriptions of guarding patterns b. Replacement of sentinels”, the authors state in the last line: “The conflict between the two was often manifested by both of them nervously preening themselves”. How do we know there was conflict?
5. Under the heading “Descriptions of guarding patterns c. Allofeeding”, the authors state in the end “… suggesting that despite being hungry it had refused to accept the “gift” from another babbler”. What is the interpretation of refusing to accept food despite being hungry, especially for a subordinate? Please elaborate.
6. In Fig. 2, there is no mention of F1.
7. In all the figures, the colour contrast between different bars is very poor.

I have read this submission. I believe that I have an appropriate level of expertise to confirm that it is of an acceptable scientific standard, however I have significant reservations, as outlined above.

# Response

**Published:** 2016-01-11

**Authors:** Amotz Zahavi

**Affiliations:** Tel Aviv University, Israel

1–Q.  If the authors wish to argue that altruistic acts are not actually altruistic, they should not continue to say “altruistic act of guarding”. Perhaps they should something like “supposedly altruistic act of guarding”.

Answer: Although altruism is defined by biologists as an act that helps others at a cost in fitness to the altruist, this is not the only definition of the term. Ordinary dictionaries define altruism as an investment that helps others. It is not dependent on whether or not the fitness of the altruist is reduced as a result, consequently, guarding when the rest of the group is feeding is indeed an altruistic act.

We have repeatedly suggested that, on average, the direct fitness of the altruist is increased, and that "This may explain why altruistic acts are so common among animals living in cooperative groups."  See also: "Altruism and moral behaviour." (page 149, in Zahavi A, Zahavi A: The Handicap Principle).

In a more recent paper (~~Zahavi, A. and Zahavi, A. (2012). The logic of analog signaling and the theory of signal selection. Israel Journal of Ecology & Evolution. 58, 269-278) we suggest use of the term "investment" rather than "cost" because observers of altruistic acts can evaluate the investment of the altruist; whereas, when the investment is described as "cost" this already implies that it reduces the fitness of the altruist which is not necessarily the case

2. Description of Guarding. Our "questionable sentence" is preceded by a description of sentinel activity. This description does not differ from that used in almost every study dealing with sentinel activity

3. This is a short way of writing: "Food availability has a profound effect:  On a rainy day (for instance, Feb 22 in figure 2 , supplementary material) little guarding was observed -  because it was difficult to find food while on the following day (for instance Feb 23, in the same figure) a maximum guarding duration of almost 45 minutes/h was reached, probably due to the availability of a large number of insects that had drowned in the flooding, facilitating spending less time on looking for food and more time on guarding"

4. See the attached video clip.

5. If allofeeding may be considered an expression of dominance (Woolfenden and Fitzpatrick (1977) Kemp and Kemp (1980), Kalishov Zahavi and Zahavi, (2005)), then refusing to be fed reduces the prestige of the donor and increases that of the receiver. The fact that the recipient came to us to get food showed that it had not refused the food from the would-be donor because it was satiated.

# Peer review: Referee response for version 1

**DOI:** 10.5256/f1000research.7238.r10649

**Published:** 2015-10-02

**Authors:** Stephen Rothstein

**Affiliations:** 1 Department of Ecology, Evolution and Marine Biology, University of California, Santa Barbara, Santa Barbara, CA, USA

This paper reports observations of guarding in Arabian babblers in considerable detail and does an excellent job of relating various aspects of guarding to variables such as a bird’s dominance rank and its manner of replacing an individual who is already guarding.  The results are new and important and the paper convincingly shows what looks like an element of competition in terms of who guards.  This is a fascinating and valuable result which suggests that individuals that guard receive some increase in fitness from their guarding behavior.  The paper argues that this fitness increment is via an increase in an individual’s prestige because time spent guarding is a handicap that takes away from time that could be spent feeding and because guarding may increase the guarding individual’s risk of predation.  All of this train of logic holds together although I am not sure there is much actual evidence for the existence of prestige and its assumed effect on fitness.

A problem as I see it is that there may be alternate explanations for the reason that dominant individuals seem to go out of their way to guard and even displace individuals that are already guarding. First, the alpha male may guard the most because he has the most to gain from guarding as he has fathered the offspring produced in the territory. Second, the assumption seems to be that guarding is done to reduce predation.  But what if it is also done to repel conspecific intruders, especially males that might try and mate with the breeding female? The individual that would gain the most from repelling conspecific intruders is the alpha male and this could explain why alpha males do most of the guarding and even displace subordinate males from guarding as the latter would have less to gain from taking action against conspecific intruders. Guarding against intruding rival males explains why alpha males greatly increase guarding during the courtship because that is when extra-pair copulations would be most costly to their fitness. I did not see any clear explanation for this change in alpha males’ guarding under the  prestige explanation.  Guarding against intruding rival males could also explain why so many alpha male guarding sessions start with a direct displacement of another guarding bird as intruders are not as much of a threat (or any threat) to the fitness of other group members. Alpha males may sometimes allofeed birds already guarding because that may ease the transition and allofeeding may occur when an alpha males has found abundant food and is already satiated. Group members other than the alpha male may engage in guarding because they may eventually become the dominant male or female, i.e. they may benefit from maintaining the integrity of the group territory.

If demonstrating to other group members a male’s ability to engage in seemingly altruistic acts is an important aspect of guarding, then alpha males should be more likely to initiate a guarding session when another group member is already guarding than when no one is guarding. The authors seem to have the data to test that prediction.  The issue is admittedly complicated because there might be a greater need to guard against predators when no one else is guarding than when someone else is already guarding.  But the relevant analysis might still be worth doing because it does have the potential to support the authors’ line of reasoning.

There is no doubt that the babbler system is a very complicated one and that the Zahavis’ long term observations have made this into a classic system that has been very valuable to research on avian breeding and behavioral ecology in general. This paper adds to the novelty of the babbler system and it is only the final bit of the authors’ logic stream, that individuals incur a handicap so as to demonstrate their prowess and accrue prestige that remains unconvincing to me. More attention to alternative hypotheses and to testing these against the favored hypothesis might in the end provide more convincing support for the latter.

I have read this submission. I believe that I have an appropriate level of expertise to confirm that it is of an acceptable scientific standard.

# Response

**Published:** 2016-01-11

**Authors:** Amotz Zahavi

**Affiliations:** Tel Aviv University, Israel

Q.---First, the alpha male may guard the most because he has the most to gain from guarding as he has fathered the offspring produced in the territory.

Second, the assumption seems to be that guarding is done to reduce predation. But what if it is also done to repel conspecific intruders, especially males that might try and mate with the breeding female? The individual that would gain the most from repelling conspecific intruders is the alpha male and this could explain why alpha males do most of the guarding and even displace subordinate males from guarding as the latter would have less to gain from taking action against conspecific intruders

Answer -There is no doubt that the detection of conspecifics is one of the most important aims of guarding. However, the idea that the subordinate male would have less to lose if the territory is taken over is incorrect, because when alien males or females take over the group in order to breed, all the members of the same sex as the alien in the original group (except sometimes the very young fledglings) are chased away (Zahavi A: (1990). Arabian Babblers: The quest for social status in a cooperative breeder. In Stacey PB and Koenig WD editors. Cooperative breeding in birds. Long-term studies of ecology and behavior. Cambridge: Cambridge University press. 1990; 103–130). The expelled individuals become refugees. It is therefore in the interest of every individual in the group to insure that intruders of the same sex will not take-over the group.

Q .---Extra-pair copulations. The referee suggests that guarding against intruding rival males explains why alpha males greatly increase guarding during the courtship because that is when extra-pair copulations would be most costly to their fitness. "I did not see any clear explanation for this change in alpha males’ guarding under the prestige explanation."

Answer. Breeding females do not copulate with males of neighbouring groups, but may copulate with subordinate males of their own group that are not their offspring. Such extra-pair copulations are indeed of great concern for the alpha male.  –Mate-guarding by the alpha male is aimed against copulations by the beta or lower ranking males that are not the descendants of the breeding female. This is why competition among same-sex members of the group increases during the breeding season. As there is almost no aggression among the same-sex adults, the competition is expressed mainly by increased guarding and by allofeeding – as demonstrations of dominance.

Q.---Alpha males may sometimes allofeed birds already guarding because (1) that may ease the transition and (2) allofeeding may occur when an alpha males has found abundant food and is already satiated.

Answer.  (1) It has repeatedly been suggested that allofeeding is a demonstration of dominance (Kalishov A, Zahavi A, Zahavi A: (2005).  Allofeeding in Arabian Babblers (Turdoides squamiceps). J Ornithol. 2005; 146(2): 141–150.; Kemp AC, Kemp MI (1980). The biology of the southern ground hornbill Bucorvus leadbeateri (Vigros). Ann Transvaal Museum 32:65–100.; Woolfenden GE, Fitzpatrick JW (1977). Dominance in the Florida scrub jay. Condor 79:1–12). As such it aggravates rather than mitigates the situation. This is manifested by the recipient often trying to avoid being fed (see "refusals" in Table 4).

(2) In most cases the alpha male is not necessarily satiated when allofeeding. It often flies down to take tidbits from the observer immediately after taking up its guarding post following the allofeeding.  Similarly, the individual that has just refused to accept food from its replacer will often readily accept the same kind of food when offered by the observer. Satiated individuals usually do not approach the offered food .

# Peer review: Referee response for version 1

**DOI:** 10.5256/f1000research.7238.r10220

**Published:** 2015-09-07

**Authors:** Uzi Motro

**Affiliations:** 1 Department of Ecology, Evolution and Behavior, Hebrew University of Jerusalem, Jerusalem, Israel

A very stimulating paper, looking at the vigilance conflict in a different, original way.

The idea that taking on a social task (such as guarding the group) that inflicts an obvious burden on the performer, is not necessarily an altruistic act, has already been presented by Zahavi and their associates. Their assertion is that although such a behavior looks altruistic, indeed it is not – by accepting the burden, the performer reliably indicates its high quality, thus gaining a prestige which increases its reproductive success. The contribution of the present paper is in providing real data, which unequivocally confirm their handicap assertion.

However, I have some concern with the statistical analysis of the data. The units of sampling are, obviously, the various groups. Thus, the observations on the alpha males, on the beta males and on the females do not represent independent samples, but constitute paired observations. The correct statistical tool should be a paired-observation comparison, such as a paired *t*-test. Moreover, Bonferroni-type adjustments for multiple comparisons are needed in some places.

I took the liberty of performing paired *t*-tests on the source data (Dataset 1), and to my satisfaction, although the *p*-values have changed, the conclusions remain valid.

And a small comment: In the Introduction, the authors claim that "However, that guarding helps the group does not explain why a particular individual may forego foraging or any other occupation, in order to guard." There are several works that demonstrate how guarding can be evolutionarily stable in certain situations (see below some examples). The novelty in the present paper is that it can explain the *competition* over performing the act of guarding.

Pulliam, H. R., Pyke, G. H. & Caraco, T. (1982). The scanning behaviour of juncos : a game-theoretical approach. *Journal of Theoretical Biology *95: 89-103.

Parker, G. A. & Hammerstein, P. (1985). Game theory and animal behaviour. In: *Evolution: Essays in Honour of John Maynard Smith. *(Greenwood, P. J., Harvey, P. & Slatkin, M., eds.) pp. 73-94. New York: Cambridge University Press.

Motro, U. & Cohen, D. (1989). A note on vigilance behavior and stability against recognizable social parasites. *Journal of Theoretical Biology *136:21-25.

I have read this submission. I believe that I have an appropriate level of expertise to confirm that it is of an acceptable scientific standard, however I have significant reservations, as outlined above.

# Response

**Published:** 2016-01-11

**Authors:** Amotz Zahavi

**Affiliations:** Tel Aviv University, Israel

~~The statistical analysis was corrected using Motro's calculations. See new version.
